# Supplementary material for: Risk assessment of workers’ exposure to BTEX and hazardous area classification at gasoline stations
Source: PLoS One. 2021 Apr 15;16(4):e0249913. doi: 10.1371/journal.pone.0249913 (PMC8049477; doi:10.1371/journal.pone.0249913)
Supplement: S1 File — (PDF) [file pone.0249913.s001.pdf]

Code of station.........................

Gasoline station survey form

"Hazardous area classification of the gasoline stations in Thailand"

Part 1 - Characteristics of gasoline stations

Part 2 – Questions specific to gasoline worker's characteristics

Part 1. Characteristics of gasoline stations			
Item	Question	Guidance choices	For researcher
1	Type of retail petroleum station by Energy Ministry classification	Type A. .... 1<br>Type B. 2	Type [ ]
2	How many years has it been open for service?	number ....year<br>since	service [ ]
3	What is the daily operation time of the station?	..........1. 06.00 – 22.00 hr.<br>..........2. 24 hr.<br>..........3. Other	Operatetime [ ]
4	How many gasoline workers are there?	amount ....persons	Employees [ ]
5	How many shiftwork per day are there on the service?	amount .... shift	Shift [ ]
6	How many workers are there on each shiftwork?	amount .... workers/shift	Shiftstaff [ ]
7	How many fuel dispensers are there?	amount .... dispenser	dispenser [ ]
8	How many nozzles per fuel type are there; please identify.	Benzine .... nozzles<br>gasohol 95 .... nozzles<br>gasohol 91 .... nozzles<br>gasohol E20 .... nozzles<br>gasohol E85 .... nozzles<br>Diesel .... nozzles<br>Other .... nozzles	Ben_noz [ ]<br>Gas95_noz [ ]<br>Gas91_noz [ ]<br>Gas20_noz [ ]<br>E85_noz [ ]<br>Des_noz [ ]<br>Other_noz [ ]
9	Has a vapor recovery system (VRS) been installed on the fuel nozzle?	No ....0 (move to item 11)<br>Yes 1	VRS [ ]
10	How many VRS nozzles are there?	amount .... nozzles	VRS_num [ ]
11	How many liters of gasoline are sold daily in total?	amount .... liters	sold_vol [ ]
12	How many liters of each gasoline type are sold daily?	benzene .... liters<br>gasohol 95 .... liters<br>gasohol 91 .... liters<br>gasohol E20 .... liters<br>gasohol E85 .... liters<br>Diesel .... liters<br>Other .... liters	Ben_vol [ ]<br>Gas95_vol [ ]<br>Gas91_vol [ ]<br>Gas20_ vol [ ]<br>E85_ vol [ ]<br>Des_ vol [ ]<br>Other_ vol [ ]

13	What is the safety distance of the following in meters?;<br>- safety distance setback from the main road	.... 1. < 5 meters<br>.....2. ≥ 5 meters	stbacrod [ ]
14	- safety distance between the fuel storage tank and service building	...... 1. < 5 meters<br>...... 2. ≥ 5 meters	sttakbud [ ]
15	- safety distance between the fuel storage tank and fuel dispenser	...... 1. < 5 meters<br>...... 2. ≥ 5 meters	sttakdisp [ ]
16	- safety distance between each fuel dispenser	...... 1. < 5 meters<br>........2. ≥ 5 meters	stdisp2 [ ]
17	- safety distance between fuel dispensers and the service building	........1. < 5 meters<br>........2. ≥ 5 meters	stdispbud [ ]
18	Service type classification:<br>Type IV; fuel dispenser house, oil storage tanks, office, maintenance store minimart, coffee shop, food court<br>Type III; fuel dispenser house, oil storage tanks, office, maintenance store minimart, coffee shop<br>Type II; fuel dispenser house, oil storage tanks, office, maintenance store<br>Type I; fuel dispenser house, oil storage tanks, office	...... 1. Type I<br>...... 2. Type II<br>...... 3. Type III<br>...... 4. Type IV	service [ ]
**Part 2: Questions specific to a gasoline worker's characteristics**			
19	Age	..........years.......month	Age [ ]
20	What is your main service function at the gasoline station?	........1. Refueling service<br>........2. Cashier	function [ ]
21	How many years of experience do you have working at the gasoline station	.... years<br>............months	exper [ ]
22	How many days per week do you usually work at the station?	...........days/week	day [ ]
23	How many hours per day do you usually work at the station?	.... hours/day	hours [ ]
