# Supplementary material for: Risk assessment of workers’ exposure to BTEX and hazardous area classification at gasoline stations
Source: PLoS One. 2021 Apr 15;16(4):e0249913. doi: 10.1371/journal.pone.0249913 (PMC8049477; doi:10.1371/journal.pone.0249913)
Supplement: S2 File — (PDF) [file pone.0249913.s002.pdf]

รหัสสถานีบริการน้ำมันเชื้อเพลิง..................................

<u>แบบสำรวจสถานีบริการจำหน่ายน้ำมันเชื้อเพลิง</u>

"การจำแนกพื้นที่อันตรายของสถานีบริการจำหน่ายน้ำมันเชื้อเพลิงในประเทศไทย"

ข้อมูลประกอบด้วย 2 ส่วน

ส่วนที่ 1 ข้อมูลทั่วไปของสถานีบริการน้ำมันเชื้อเพลิง

ส่วนที่ 2 ข้อมูลของพนักงานสถานีบริการน้ำมันเชื้อเพลิง

ส่วนที่ 1 ข้อมูลทั่วไปของสถานีบริการน้ำมันเชื้อเพลิง			
**ข้อที่**	**คำถาม**	**แนวทางตัวเลือก / คำตอบ**	**สำหรับผู้วิจัย**
1	ประเภทสถานีบริการน้ำมันเชื้อเพลิง จำแนกตามประกาศของกระทรวงพลังงาน	...........1. ประเภท ก.<br>...........2. ประเภท ข.	Type [ ]
2	ปัจจุบันสถานีบริการฯ เปิดบริการมากี่ปี	จำนวน .... ปี<br>เปิดตั้งแต่ปี	service [ ]
3	สถานีบริการฯ เปิดให้บริการเวลาใด	.... 1. 06.00 – 22.00 น.<br>........2. 24 ชม.<br>....... 3. อื่นๆ	Operatetime [ ]
4	ปัจจุบันมีจำนวนพนักงานเท่าไหร่	จำนวน .... คน	Employees [ ]
5	สถานีบริการฯ ทำงานกี่กะ	จำนวน .... กะ	Shift [ ]
6	สถานีบริการฯ ทำงานกะละกี่คน	จำนวน .... คนต่อกะ	Shiftstaff [ ]
7	สถานีบริการฯ มีหัวจ่ายน้ำมันเชื้อเพลิงเท่าไหร่	จำนวน .... หัวจ่าย	dispenser [ ]
8	จำนวนหัวจ่ายน้ำมันเชื้อเพลิงแต่ละชนิดในสถานีบริการฯ	เบนซิน .... หัวจ่าย<br>โซฮอล์ 95.............. หัวจ่าย<br>โซฮอล์ 91.............. หัวจ่าย<br>โซฮอล์ E20 ....หัวจ่าย<br>E 85 .... หัวจ่าย<br>ดีเซล ....หัวจ่าย<br>อื่นๆ ....หัวจ่าย	Ben_noz [ ]<br>Gas95_noz [ ]<br>Gas91_noz [ ]<br>Gas20_noz [ ]<br>E85_noz [ ]<br>Des_noz [ ]<br>Other_noz [ ]
9	สถานีบริการฯ ระบบหัวจ่ายน้ำมันเชื้อเพลิงแบบระบบดูดไอน้ำมันกลับ (VRS) หรือไม่	........0. ไม่มี (ข้ามไปข้อ 11)<br>........1. มี	VRS [ ]
10	จำนวนหัวจ่ายน้ำมันเชื้อเพลิงแบบระบบดูดไอน้ำมันกลับ (VRS)	จำนวน................หัวจ่าย	VRS_num [ ]
11	ปริมาณยอดขายน้ำมันเชื้อเพลิงต่อวัน (หน่วยเป็นลิตร)	จำนวน ....ลิตร	sold_vol [ ]
12	ปริมาณยอดขายน้ำมันเชื้อเพลิงแยกตามชนิดของน้ำมันเชื้อเพลิงตามรายวัน (หน่วยเป็นลิตร)	เบนซิน .... หัวจ่าย<br>โซฮอล์ 95.............. หัวจ่าย<br>โซฮอล์ 91.............. หัวจ่าย<br>โซฮอล์ E20 .... หัวจ่าย<br>E 85 .... หัวจ่าย<br>ดีเซล .... หัวจ่าย<br>อื่นๆ .... หัวจ่าย	Ben_vol [ ]<br>Gas95_vol [ ]<br>Gas91_vol [ ]<br>Gas20_ vol [ ]<br>E85_ vol [ ]<br>Des_ vol [ ]<br>Other_ vol [ ]
13	ระยะห่างปลอดภัยสำหรับเขตสถานีบริการและถนนสาธารณะ	.......1. น้อยกว่า 5 ม.<br>......2. มากกว่าหรือเท่ากับ 5 ม.	stbacrod [ ]
14	ระยะห่างปลอดภัยระหว่าง ถังเก็บน้ำมันเชื้อเพลิงและอาคารบริการ	.......1. น้อยกว่า 5 ม.	sttakbud [ ]

		......2. มากกว่าหรือเท่ากับ 5 ม.	
15	ระยะห่างปลอดภัยระหว่าง ถังเก็บน้ำมันเชื้อเพลิง และแท่นหัวจ่ายน้ำมันเชื้อเพลิง	......1. น้อยกว่า 5 ม.<br>......2. มากกว่าหรือเท่ากับ 5 ม.	sttakdisp [ ]
16	ระยะห่างปลอดภัยระหว่าง แต่ละแท่นหัวจ่ายน้ำมันเชื้อเพลิง	......1. น้อยกว่า 5 ม.<br>......2. มากกว่าหรือเท่ากับ 5 ม.	Stdisp2 [ ]
17	ระยะห่างปลอดภัยระหว่าง แท่นหัวจ่ายน้ำมันเชื้อเพลิงและอาคารบริการ	......1. น้อยกว่า 5 ม.<br>......2. มากกว่าหรือเท่ากับ 5 ม.	stdispbud [ ]
18	ประเภทของสถานีบริการฯ จำแนกตามชนิดการจัดบริการ<br>Type IV = อาคารหัวจ่ายน้ำมัน, ถังเก็บน้ำมัน, สำนักงาน, ร้านซ่อมบำรุงรถยนต์, ร้านค้าสะดวกซื้อ, ร้านกาแฟ, ร้านบริการอาหาร<br>Type III = อาคารหัวจ่ายน้ำมัน, ถังเก็บน้ำมัน, สำนักงาน, ร้านซ่อมบำรุงรถยนต์, ร้านค้าสะดวกซื้อ, ร้านกาแฟ<br>Type II = อาคารหัวจ่ายน้ำมัน, ถังเก็บน้ำมัน, สำนักงาน, ร้านซ่อมบำรุงรถยนต์<br>Type I = อาคารหัวจ่ายน้ำมัน, ถังเก็บน้ำมัน, สำนักงาน	...... 1. Type I<br>...... 2. Type II<br>...... 3. Type III<br>...... 4. Type IV	service [ ]

ส่วนที่ 2 ข้อมูลของพนักงานสถานีบริการน้ำมันจำหน่ายน้ำมัน			
19	อายุ (ปี)	.............ปี	Age [ ]
20	หน้าที่หลักหรือตำแหน่งงาน	.............พนักงานเติมน้ำมัน<br>.............พนักงานเก็บเงิน	function [ ]
21	ประสบการณ์การทำงานในสถานีบริการจำหน่ายน้ำมันเชื้อเพลิงแห่งนี้	.... ปี<br>................เดือน	exper [ ]
22	ทำงานในสถานีฯ นี้กี่วันต่อสัปดาห์	.... วัน/สัปดาห์	day [ ]
23	ทำงานในสถานีฯ กี่ชั่วโมงต่อวัน	.... ชั่วโมงต่อวัน	hours [ ]
