# Supplementary material for: Risk assessment of workers’ exposure to BTEX and hazardous area classification at gasoline stations
Source: PLoS One. 2021 Apr 15;16(4):e0249913. doi: 10.1371/journal.pone.0249913 (PMC8049477; doi:10.1371/journal.pone.0249913)
Supplement: Attachment — Submitted filename: Response to Reviewers -170321.docx [file pone.0249913.s003.docx]

PONE-D-20-27491
Risk assessment on workers’ exposure to BTEX and hazardous area classification at gasoline stations


Dear PLOS ONE Academic Editor,

According to the comments of reviewers and editor, we author appreciated all suggestions to improve the paper presentation and all correction and clarifications are the following;

| Reviewer | Comments | Answers and additional comments | Location |
| --- | --- | --- | --- |
| **Academic Editor** | Ensure that your manuscript meets PLOS ONE's style requirements, including those for file naming | Format was rechecked and approved |  |
|  | Include additional information regarding the survey or questionnaire used in the study and ensure that you have provided sufficient details that others could replicate the analyses. | Questionnaire was included to one uploaded attached file; Gasoline station data survey form |  |
|  | Remove any funding-related text from the manuscript | Financial disclosure was deleted from an acknowledgement, and updated the funding statement |  |
|  | provide more information (and updated) on the toxicological aspects of BTEX compounds | Added more referenced of Thai gasoline workers exceeded the acceptable limit [5,7,8] and similar to the studies in Malaysia [9, 10] | on comment of reviewer #2 |
|  | required about on the quality controls undertaken in the laboratory for the analysis of the samples | QC and analysis of samples were edited to provide more information in the methodology section. | Data collection;  A flammable gas detector was used to measure the total VOCs emission (Photo ionization detector (PID) sensor (detection range of 0 to 1000 ppm) and the lower explosive limit - upper explosive limit (LEL-UEL) with Non-Dispersive Infrared (NDIR) combustible sensors (range of 0-100%LEL) |
|  | when the samples were collected. | Sample collected in the season was stated | Data collection;  The samples were collected between June and July 2018 June and July 2018, at times when the temperature was 28**.**08 ±1**.**58 °C, humidity was 78**.**51±8**.**89%, and wind velocity was 10.0 ±2**.**82 m**/**min |
| **Reviewer #1** | 1. Title: Risk assessment "of" workers´ exposure (delete "on"). | Revised; Title: Risk assessment "of" workers´ exposure (delete "on"). | Title ..of… |
|  | 2. Lines 31-32. "radius around the fuel dispensers" (delete "in") | Revised; “radius around the fuel dispensers” | Abstract;  The risk matrix classified …radius around the fuel dispensers |
|  | 3. Line 93-94: "as described in NIOSH method 1501 [15]" delete "which followed NIOSH number 1501 [15] methodology | Revised; "as described in NIOSH method 1501 [15]" | Data collection;  The BTEX concentrations were measured… as described in NIOSH method 1501… |
|  | 4. Line 95: "monitored" not "monitoring" | Revised; "monitored" | Data collection;  The exposure to BTEX was monitored |
|  | 5. Line 103: describe the acronym LEL-UEL | Revised; “describe the acronym LEL-UEL “ | Data collection;  A flammable gas detector … lower explosive limit - upper explosive limit (LEL-UEL)… |
|  | 6. Line 171-176: the terms Ox, Oy are not represented in the formula. It is not clear what terms in the formula the refer to. | Revised; Changed the symbol “σ_y_, σ_z_” to match the equation. | Hazardous area classification at gasoline stations;  The BTEX_emiss_ concentrations at different distances were σ_y_ σ_z_ .. |
| **Reviewer #2** | 1. Abstract: Include the full name of EPA-IRIS | Revised; Added full name of EPA-IRIS in abstract (however, it caused abstract to be more than 250 word). | Abstract;  The risk assessment of gasoline workers at … US. Environmental Protection Agency-IRIS |
|  | 2. Safe standard of benzene (100 ppb): Include name of the agency on country suggest the value of concentration. | Revised; “NIOSH exposure limits of 100 ppb benzene concentration” | Abstract;  Results showed that the BTEX concentrations… of the NIOSH exposure limit of 100 ppb of benzene concentration. |
|  | 3. In term of comparison with previous studies, I suggested the author include previous similar studies conducted in Thailand | Revised; comparison with previous studies in introduction and discussion. | Introduction;  It was also shown that the lifetime cancer risk…8]  Discussion;  …and was higher than the previous finding in Khon Kaen, Thailand [12] |
|  | 4. In term comparison with other countries I suggest the author to consider similar studies conducted in Southeast Asian countries which have similar weather condition | Revised; comparison with previous studies in Southeast Asian countries in introduction and discussion. | Introduction;  It was also shown that the lifetime cancer risk… and similar to the studies in Malaysia [9, 10].  Discussion;  .. like in gasoline station of Kuwait study [15]. The finding of concentration at gasoline station in Malaysia [9] was quite lower or closed to the range in our study. |
|  | 5. Provide detail information on BTEX sampling procedures. What is the specific time for 4 hours of BTEX measurement? Provide information on replications and QA/QC including the method detection limit for BTEX measurement. | Revised;  1. the information of 4 hours measurement was calculated to be 8 hour exposure to compare to the standard limit of working period, which were added to the method.  2. Added the limit of detection (LOD) of all BTEX  and the VOCs measured by gas detector | Data collection;  1. The exposure to BTEX was monitored… covered the high service hours for 4 hours sampling.  2. Each tube was extracted with… and limit of detection (LOD) was < 0.001 ppm and < 0.003 ppb for toluene and benzene and < 0.05 ppb for ethyl benzene and xylene.  ……measure the total VOCs emission (Photo ionization detector (PID) sensor (detection range of 0 to 1000 ppm) and the lower explosive limit - upper explosive limit (LEL-UEL) with Non-Dispersive Infrared (NDIR) combustible sensors (range of 0-100%LEL). |
|  | 6. The characteristics of sampling stations can be included in the Methodology section rather than the Results section. | Revised;  - Added sample inclusion criteria; gasoline dispenser nozzles, and gasoline stations operation time in the methodology section. | Sample size;  There were two inclusion criteria… and gasoline dispenser nozzles; and 3) they had to have more than 8 hours of daily operation time. |
|  | 7. Compare the concentration of BTEX record in this study to previous similar studies | Revised;  1. BTEX comparison on in discussion section.  2. HQ and HI comparison in discussion | Discussion  1. BTEX concentrations in the ambient… and accessed of the NIOSH standard level (100 ppb) like in gasoline station of Kuwait study [15]. The finding of concentration at gasoline station in Malaysia [9] was quite lower or closed to the range in our study.  2. From the adverse effect risk… in Thailand [6] and Malaysia [9, 10], where had the similar climatic conditions to Thailand. The study showed that… study [5] as confirmed by this study and the characteristic of a function as refueling service working closed to the gasoline dispensers could exhibit the higher health risk [5]. |
